# Motor control drives visual bodily judgements

**DOI:** 10.1016/j.cognition.2019.104120

**Published:** 2020-03

**Authors:** Roni O. Maimon-Mor, Hunter R. Schone, Rani Moran, Peter Brugger, Tamar R. Makin

**Affiliations:** a Institute of Cognitive Neuroscience, University College London, London WC1N 3AZ, UK; b WIN Centre, Nuffield Department of Clinical Neuroscience, University of Oxford, Headington, Oxford OX3 9DU, UK; c Max Planck University College London Centre for Computational Psychiatry and Ageing Research, University College London, London WC1B 5EH, UK; d Department of Neurology, Neuropsychology Unit, University Hospital Zurich, Switzerland

**Keywords:** Amputees, Visuomotor, Body representation, Phantom limb, Motor simulation, Embodied cognition

## Abstract

The ‘embodied cognition’ framework proposes that our motor repertoire shapes visual perception and cognition. But recent studies showing normal visual body representation in individuals born without hands challenges the contribution of motor control on visual body representation. Here, we studied hand laterality judgements in three groups with fundamentally different visual and motor hand experiences: two-handed controls, one-handers born without a hand (congenital one-handers) and one-handers with an acquired amputation (amputees). Congenital one-handers, lacking both motor and first-person visual information of their missing hand, diverged in their performance from the other groups, exhibiting more errors for their intact hand and slower reaction-times for challenging hand postures. Amputees, who have lingering non-visual motor control of their missing (phantom) hand, performed the task similarly to controls. Amputees’ reaction-times for visual laterality judgements correlated positively with their phantom hand’s motor control, such that deteriorated motor control associated with slower visual laterality judgements. Finally, we have implemented a computational simulation to describe how a mechanism that utilises a single hand representation in congenital one-handers as opposed to two in controls, could replicate our empirical results. Together, our findings demonstrate that motor control is a driver in making visual bodily judgments.

## 1 Introduction

Converging evidence suggests that our sensorimotor body experiences affect the way visual information is interpreted (Hagura, Haggard, & Diedrichsen, 2017; Makin, Wilf, Schwartz, & Zohary, 2010; van den Heiligenberg, Yeung, Brugger, Culham, & Makin, 2017) and even processed (Aglioti, Cesari, Romani, & Urgesi, 2008; Maimon-Mor, Johansen-Berg, & Makin, 2017). According to this view, also known as ‘embodied cognition’ (Wilson, 2002), our bodily interactions with the environment, and motor control in particular, may play a fundamental role in the development of our perceptual and cognitive abilities. If the theory of embodied cognition is verified, any attempt to accurately describe perception and cognition (e.g. developing artificial models of these processes), should consider visuomotor processing as a bidirectional process.

The most compelling evidence for the role of motor control in driving visual processing comes from visual body representation, and hand representation in particular. Historically, performance on visual hand laterality judgement tasks was thought to reflect sensorimotor processes (e.g. implicit motor imagery). This was largely due to evidence showing that task performance when making hand laterality judgements reflected some of the postural biomechanical complexity of the hand stimuli. For example, participants take longer to identify the laterality of more physically awkward (but not visually complex) hand postures (1994, Cooper & Shepard, 1975; Parsons, 1987; Sekiyama, 1982). This link between implicit motor imagery and laterality performance has been further reinforced by evidence demonstrating that performance is impacted by handedness (Gentilucci, Daprati, & Gangitano, 1998; Ní Choisdealbha, Brady, & Maguinness, 2011; Parsons, 1987, 1994), hand position held during the task (Ionta, Fourkas, Fiorio, & Aglioti, 2007; Shenton, Schwoebel, & Coslett, 2004) and atypical motor control (Conson, Pistoia, Sarà, Grossi, & Trojano, 2010; Conson et al., 2013; Fiorio, Tinazzi, & Aglioti, 2006; Helmich, de Lange, Bloem, & Toni, 2007). Collectively, this corpus of evidence has been interpreted as a powerful demonstration of embodied visual cognition.

More recently, the notion that motor experience is fundamental for visual body perception has been challenged by studies performed in one to five individuals with congenital absence of both hands (Vannuscorps & Caramazza, 2015, 2016; Vannuscorps, Pillon, & Andres, 2012). Despite not having any motor hand experience, visual hand perception (Vannuscorps & Caramazza, 2016), representation (Striem-Amit, Vannuscorps, & Caramazza, 2017) and judgements (Vannuscorps et al., 2012) were reported to not differ from those of typically developed two-handed controls. It has therefore been suggested that the ability to process visual body information is either innate or depends on passive (3^rd^ person) visual experience accumulated by observing others, and can therefore be entirely decoupled from motor experience. Because first-person motor experience is inherently coupled with visual experience, this new account highlights the fundamental role of visual experience in informing visual laterality judgements and puts into question the historical interpretation for motor-related visual body representation. For example, hand configurations which are mechanically less common/plausible will also be viewed less frequently; patients with reduced motor abilities would also suffer reduced visual experience with the affected hand due to reduced usage in daily life, etc. As such, it is currently unknown whether motor experience informs visual body representation.

**Fig. 1:**
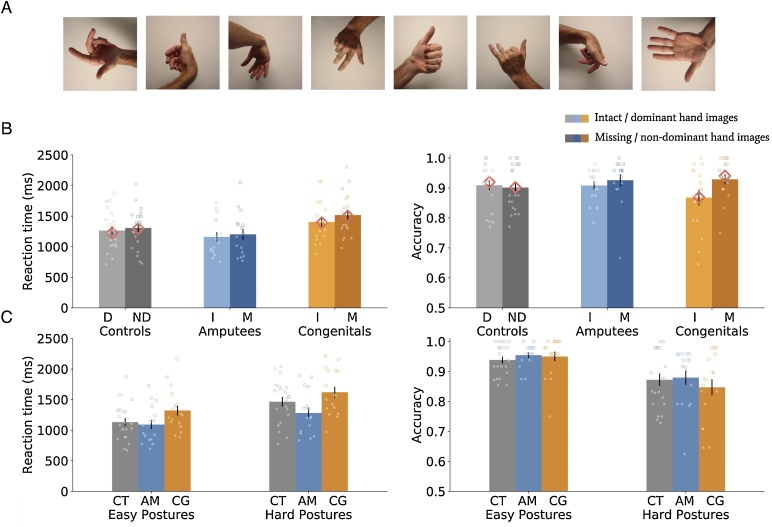
Hand laterality judgment stimuli and results. (A) Example stimuli used in the hand laterality judgement task. (B) Group performance (absolute RT, left; Accuracy, right) in the hand laterality judgement task is shown for controls (grey), amputees (blue) and congenital one-handers (orange) for the intact and missing hands (light vs dark shades, respectively). Dots correspond to individual performance. (C) Group performance (absolute RT, left; Accuracy, right) in the hand laterality judgement task is shown for easy and hard postures in controls (grey), amputees (blue) and congenital one-handers (orange). Values indicate means ± standard error. Congenital one-handers exhibit slower RTs in hard postures compared to controls. Congenital one-handers, but not amputees and controls, also show an accuracy difference between the two hands. CT = controls; AM = amputees and CG = congenital one-handers. For RT, absolute RT values are plotted, however all statistical analyses were performed on log-transformed RT values (see supplementary figure S5 for plots with log-transformed RT values). Pink diamonds depict the predicted performance values from the computational model. D = dominant side images and ND = non-dominant side images. I = intact side images and M = missing side images (For interpretation of the references to colour in this figure legend, the reader is referred to the web version of this article).

While passive visual experience alone might be sufficient to capture the approximate biomechanical constraints of a human hand, when judging more complex postures involving atypical finger and wrist orientations, individuals may increase their reliance on their first-person experience, and in particular their motor control. To determine whether motor or visual experience is integral in more challenging decision making, we tested laterality judgements of images of hand postures with varying degrees of biomechanical complexity (Moseley, 2004) in three groups of individuals, with similar passive visual experience, but different amounts of active (1^st^ person) visual and motor control (both past and present; see Table 1): (i) two-handed *controls* (n = 21), (ii) individuals born with one hand, due to a transverse deficiency (hereafter *congenital one-handers;* n = 17), and (iii) individuals who lost one hand due to amputation, with varying degrees of phantom motor abilities (hereafter *amputees;* n = 16; see Table 2 for demographic and clinical details). Participants were asked to verbally indicate whether the observed laterality of a given hand posture is left or right (See Fig. 1A for sample stimuli and Figure S1 for the complete stimuli set and classification).

**Table 1:** Summary of the visual and motor experiences across groups. Group similarities/differences in passive/other (3^rd^ person) visual experience, active/self (1^st^ person) visual experience and motor control of their secondary hand (missing/nondominant in one/two handers respectively). While all groups have similar passive visual experience for seeing the hand of others, one-handers’ active (self) visual and motor experience of the missing hand differ from controls. Amputees, experiencing phantom sensations, also differ in current motor experience from congenital one-handers (indicated by a crossed check mark as individuals vary in the amount of phantom motor control).

Secondary Hand Exp.			
Group	Passive Visual Experience	Active Visual Experience	Motor Control
Controls (n = 21) [Two-handers]			
Congenital (n = 17) [One-handers]			
Amputees (n = 16) [One-handers]		Past:	Past:
		Phantom:	Phantom:

**Table 2:** Demographic and clinical details for the amputees and congenital one-handers. M/F = Male/Female; NA = not available. For ‘Missing hand side’, asterisk (*) = amputees that had their dominant hand amputated. Phantom motor control is measured as the average time it took participants to complete five finger-thumb opposition cycles with their phantom hand.

Subject	Gender	Age	Cause of limb loss	Age at Amputation	Level of Amputation	Missing hand side	Phantom motor control (seconds)
Amp01	F	50	Tumour	45	Above elbow	Left*	18.06
Amp02	M	57	Trauma	20	Below elbow	Left*	22.06
Amp03	M	59	Trauma	40	Above elbow	Left*	21.19
Amp04	M	58	Trauma	27	Above elbow	Left	19.02
Amp05	M	53	Trauma	28	Below elbow	Left	9.14
Amp06	M	41	Trauma	27	Above elbow	Right	19.07
Amp07	M	48	Trauma	17	Above elbow	Left	13.12
Amp08	M	37	Trauma	27	Above elbow	Left	30.23
Amp09	F	46	Trauma	38	Below elbow	Left	64.08
Amp10	M	64	Trauma	33	Below elbow	Right*	20.14
Amp11	F	24	Trauma	18	Below elbow	Right	19.02
Amp12	M	49	Trauma	37	Above elbow	Left	NA
Amp13	M	29	Trauma	24	At shoulder	Left	23.01
Amp14	M	25	Trauma	18	At wrist	Left	10.04
Amp15	M	45	Trauma	20	Below elbow	Right	24.15
Amp16	M	32	Trauma	31	Above elbow	Left	230.11
Cong01	F	49	Congenital	0	Below elbow	Left	―
Cong02	F	52	Congenital	0	Below elbow	Right	―
Cong03	M	52	Congenital	0	At wrist	Left	―
Cong04	F	25	Congenital	0	At wrist	Right	―
Cong05	M	49	Congenital	0	Above elbow	Left	―
Cong06	F	28	Congenital	0	At wrist	Left	―
Cong07	M	38	Congenital	0	Below elbow	Left	―
Cong08	F	27	Congenital	0	Below elbow	Left	―
Cong09	M	60	Congenital	0	At wrist	Left	―
Cong10	F	34	Congenital	0	Below elbow	Right	―
Cong11	F	36	Congenital	0	Below elbow	Right	―
Cong12	F	41	Congenital	0	Below elbow	Left	―
Cong13	F	61	Congenital	0	At wrist	Left	―
Cong14	M	25	Congenital	0	Below elbow	Left	―
Cong15	M	34	Congenital	0	At wrist	Left	―
Cong16	M	38	Congenital	0	Below elbow	Left	―
Cong17	F	49	Congenital	0	At wrist	Left	―

If passive viewing is sufficient to construct visual body representation, all groups should perform the task equally well. If active visual and/or motor experience is essential for complete and efficient hand representation, we expect congenital one-handers to show impaired performance in the task relative to the other groups. Never having any experience with their missing (secondary) hand, they will be limited to the motor resources of their intact hand.

Amputees, who previously had a complete representation for their now missing hand, will allow us to further test whether bodily judgments rely on past visuomotor experience or current motor control. Crucially, amputees report experiencing varying degrees of phantom sensations, and in particular individuals vary in their sense of motor control over their phantom hand (i.e. sense of kinaesthesia when asked to move their phantom hand and fingers; Kikkert et al., 2017; Raffin, Mattout, Reilly, & Giraux, 2012; Reilly, Mercier, Schieber, & Sirigu, 2006). Relevant to our purposes, this ongoing motor experience of the missing hand is decoupled from any relevant visual input of a hand. Therefore, we predict that if visual body judgments benefit from ongoing motor control, amputees will utilise the motor resources of their phantom hand to complete the visual laterality task.

## 2 Methods

Data presented in this manuscript was collected as part of a larger study, as detailed in the Open Science Framework (https://osf.io/kd2yh/). Presently, we focus on the following tasks: hand laterality judgement and motor finger tapping (described below). All the data included in the study will be made available online under the OSF listing of the study (https://osf.io/b4qks/).

### 2.1 Participants

Sixteen individuals with acquired unilateral upper-limb amputation (amputees; mean age ± s.e.m. = 44.8 ± 3, 4 with absent right-arm, 4 with a missing dominant hand, 3 females), seventeen individuals with a congenital unilateral transverse deficiency (diagnosis provided by the participants; hereafter, referred to as congenital one-handers; mean age ± s.e.m. = 41.1 ± 3, 4 with absent right-arm, 10 females) and twenty-two able-bodied controls (mean age ± s.e.m. = 40.6 ± 3, 7 left-hand dominant; 11 females) were included in the study (see Table 2 for demographic and clinical details). These participants also took part in our previous reported studies (Hahamy et al., 2017; Kikkert, Johansen-Berg, Tracey, & Makin, 2018; van den Heiligenberg et al., 2018, 2017). One control participant was excluded due to a high (27 %) number of trials without responses in the hand laterality task (range of no-response trials for other participants was 0%–10.4 %). Additionally, a secondary group of 13 able-bodied controls was used to establish a measure of image difficulty (see Supplementary Methods and Figure S1). Ethical approval was granted by Oxford University’s Medical Sciences inter-divisional research ethics committee (Ref: MSD-IDREC-C2-2014-003) and the NHS National Research Ethics Service (Ref: 10/H0707/29). Written informed consent was obtained from all participants prior to participating in the study in accordance with ethical standards established by the Declaration of Helsinki (1964).

### 2.2 Hand laterality judgement task

#### 2.2.1 Experimental procedure

All participants responded to a set of hand stimuli which included 24 unique egocentric photographs of right hands, in postures that ranged from biomechanically simple to awkward (obtained from *L. Moseley)*. These images were digitally mirrored to construct 24, otherwise identical, photographs of left hands (see example images in Fig. 1A). Participants completed two experimental blocks, each included the 48 total hand images. Hand images were presented in a random order using Presentation software (version 16.4). Participants were seated comfortably in front of a laptop computer while wearing a lapel microphone on their collar. Participants were instructed to rest their hand/s in their lap and were specifically instructed to not attempt to make any volitional movements, throughout the task. Participants responded vocally by indicating the hand laterality (left or right) of each presented image as fast as possible, while maintaining high accuracy. Each experimental trial consisted of the presentation of a hand image for a maximum of 5 s (see Fig. 1B), preceded by a 1 s fixation cross. Time from the start of the image display to voice onset was recorded as the participants’ reaction time (RT). The experimenter recorded the subject’s response (i.e. ‘right’ or ‘left’) via a keyboard press, which terminated the trial.

#### 2.2.2 Data analysis

All audio recordings and the appropriate classification of reaction-times were verified offline by a naive experimenter. Trials with noisy recordings (mean of 2.8 % of trials per participant) were excluded from further analysis. Accuracy was computed as the proportion of correct response of all valid trials. Only trials with correct responses were included in the RT analysis. To compare between the one-handed groups and controls, the missing hand was matched to the non-dominant hand of controls, and the intact hand was matched to the dominant hand of controls. For simplicity, hereafter, we will refer to the dominant hand of controls as intact, and the nondominant hand as missing.

RTs were logarithmically transformed in order to correct for the skewed RT distribution and satisfy the conditions for parametric statistical testing. Transformed RTs deviating more than 3 standard deviations from the participants’ means (separately for each condition) were removed. No more than 2 trials per participant were removed and the number of excluded trials did not differ between groups. The transformed RTs were analysed in a repeated-measures analysis of covariance (rmANCOVA; after testing for normality using the Shapiro Wilks test, p > 0.05), with a between-subject factor of group (controls/amputees/congenitals), within-subject factors of difficulty (easy/hard) and hand (intact/missing). To reduce error variance due to the large age range of our participants (25–60) and to increase statistical power (Fozard, Vercruyssen, Reynolds, Hancock, & Quilter, 1994), age was included as an a priori covariate. There were no significant interactions with age, thereby affirming homogeneity of regression slopes (as detailed in Supplementary Tables S2-S3). The measure of image difficulty was established by using data from a secondary control group (see Supplementary Methods), splitting the images to easy and hard based on the median RT of all images. Accuracy analysis was carried out using a rmANCOVA with the same conditions and covariates as described above. For both RT and accuracy data, outlier participants were identified as deviating by more than 3 standard deviations from their group mean, for each condition/group separately. Subsequently, a single outlier case was identified. ANOVA tests were repeated with the outlier excluded and reported no differences in our results.

Post-hoc comparisons of interactions were performed using paired t-tests within group when applicable or separate ANCOVA’s across groups with age as a covariate for each level of the within-group variable. Post-hoc comparisons of group effects were performed using an ANCOVA with age as a covariate for each pair of groups.

To examine biases in responses, leading to divergent profiles of errors across hands, Signal Detection Theory (Green & Swets, 1966; Stanislaw & Todorov, 1999) was used. This approach can separate a change in bias from changes in discriminability between the two hands (calculated using Lindeløv, 2011). We set up “signal” as the probability space of seeing the intact hand, therefore a positive bias value indicates a tendency to over-report “missing hand”, even when an intact hand is shown. Group differences were explored using a one-way ANOVA, with unpaired t-tests for follow-up.

### 2.3 Motor task

To assess amputees’ phantom hand motor control, a finger-thumb opposition task was used, as described and validated before (Kikkert et al., 2017). Participants were instructed to sequentially oppose each of the four fingertips to the approximated tip of their thumb, starting with the index finger. Each participant was asked to perform five repetitions of this movement cycle and to verbally indicate the end of each cycle. Participants first performed the task with their intact hand followed by their phantom hand. Emphasis was given to making (or attempting to make) ‘actual’ instead of ‘imagined’ phantom hand movements (Raffin, Giraux, & Reilly, 2012; Raffin, Mattout et al., 2012; Reilly et al., 2006). During task performance, participants were instructed to keep their eyes closed and keep other body parts still. RT was measured based on participants’ verbal reports when completing five movement repetitions. One amputee could not perform the task with their phantom limb, due to complete immobility of their phantom fingers, and was excluded from this task. As none of the congenital one-handers reported experiencing phantom-like sensations, they did not participate in this task.

### 2.4 Prosthesis usage

To assess daily prosthesis usage, we utilised a compound measure that represents both the incorporation of the prosthesis in daily activities and prosthesis wear time, based on methods described by (van den Heiligenberg et al., 2017). The Prosthesis Activity Log (PAL) is calculated from participants’ ratings on how frequently they use their prosthesis in an inventory of 27 daily activities (e.g. taking money out of wallet, zipping up a coat, etc.). To calculate prosthesis total wear time, participants rated (on a given scale) how much time they typically spend wearing their prostheses in their daily lives. Both the PAL and maximum wear-time ratings were standardized using a Z-transform and summed to create a compound prosthesis usage score. Note that PAL and prosthesis wear time ratings highly correlate with each other, as previously described by (van den Heiligenberg et al., 2018).

### 2.5 Correlation analysis

To test for the role of active visuomotor experience, amputees’ age at amputation was correlated (using a Spearman correlation) with mean RTs for images of both hands (see Results) and each hand individually (see Supplementary Figure S3). To test whether current motor control relates to performance in a visual bodily judgement task, this analysis was repeated with mean RTs of phantom hand finger-tapping. These two variables were chosen based on the study’s main research question, and in an effort to limit the number of comparisons. A non-parametric spearman correlation was used for the phantom hand motor control measure because the data did not meet assumptions of normality, as assessed using the Shapiro-Wilks test. To compare the correlation of motor control with that of active visual experience (age at amputation), we conducted a one-tailed test of the difference between two dependent correlations with one variable in common (Lee & Preacher, 2013). In addition, we also conducted exploratory analyses to account for the role of prosthesis usage, previously suggested to impact the reaction time during laterality judgements (Guo et al., 2017; Nico, Daprati, Rigal, Parsons, & Sirigu, 2004). To allow for additional post-hoc exploration of the results, the complete dataset will be available online, in the project’s OSF entry (https://osf.io/b4qks/). With that final analysis as an exception, all other analyses were performed using SPSS software (24.0).

### 2.6 Computational simulation

To explore the potential advantages of parallel bimanual processing during visual laterality judgements (see Discussion), we postulate a covert visuomotor hand posture simulator, accumulating evidence to determine the identity of the hand image stimulus. For a detailed description of the model’s algorithm and architecture see Results. Model simulation was performed using Matlab (R2017a). Hand posture simulators were based on a DDM code adapted from (Moran, Zehetleitner, Liesefeld, Müller, & Usher, 2016) with an addition of a logarithmic quit-timer.

We chose a drift diffusion model (DDM) to model the hand simulators as they have been extensively used to model cognitive processes involving two-choice decisions where evidence is accumulated over time (Ratcliff & Mckoon, 2008). While in theory a single DDM could have been used to model this task (with each boundary representing a decision threshold for one of the hands), in practice, we were unable to reproduce the response pattern observed in congenital one-handers using this approach. A start-point bias would predict a difference in accuracies accompanied by opposite differences in RTs, which does not fit the pattern we observed in the data for congenital one-handers. As such, in the present study we used two independent DDMs to simulate bimanual visuomotor representation.

The DDM component of the hand posture simulator has the following parameters: bias, threshold, drift-rate and nondecision-time. In addition, for each trial in each hand posture model, a quit-time is drawn from a logarithmic distribution with a mean of T_mu and a sigma of 1 s. The 5 parameters were manually optimised to fit the group-level RT and accuracy averages as the task was not designed for a single-subject model fit. For each group, 100,000 trials were simulated (50,000 for each hand). Averages of accuracy and RTs for correct trials are then calculated for each hand. The simulation code can be found on: https://github.com/ronimaimon/HandLateralityModel.

## 3 Results

Mean accuracies and RTs for each viewed hand (intact/missing) and group (controls, congenital one-handers, amputees) are shown in Fig. 1B and for each difficulty (easy/hard; based on performance of an independent control group on the same task) and group in Fig. 1C.

### 3.1 Accuracy

The rmANCOVA analysis of accuracy data revealed a significant interaction between hand and group (F(2,50) = 3.92, p = 0.026), we did not observe a significant main effect of hand (F(1,50) = 0.70, p = 0.41), group (F(2,50) = 0.29, p = 0.75), or a three-way interaction of group, difficulty and hand (F(2,50) = 0.86, p = 0.43; For a full description of all remaining nonsignificant results see Supplementary Table S3; for similar results using a trade-off measure RT/Accuracy, see Supplementary Table S4). These results indicate that hand-loss impacts correct task performance, particularly for the intact hand, but independent of posture difficulty (Fig. 1B&C, right). Post-hoc hand comparisons within each group confirmed that congenital one-handers show lower accuracy when judging the laterality of intact hand images compared to their missing hand (t(16) = 3.24, p = 0.005). No significant hand differences were observed for controls (t(20)=-.491, p = 0.629) or amputees (t(15) = 0.934, p = 0.365). Additionally, one extreme outlier was identified in the amputees group for hard missing hand images. Repeating the analyses above, when removing this outlier, we still find no differences to our results.

A possible intuitive explanation for this pattern of lower accuracy for the intact hand originates from the nature of the decision-making process in the task: when uncertain about the laterality of the image, congenital one-handers may prefer to guess that they are observing a non-familiar (missing hand) posture. This will not impact or slightly increase the accuracy of the missing hand, while reducing accuracy for intact hand postures. To explore this interpretation, we applied Signal Detection Theory, allowing us to dissociate between response bias (criterion) and discriminability (d’). No group differences were found in the d’ index (F(2,54) = 0.04, *p* = 0.96; see Figure S4A), indicating all groups were able to discriminate the intact hand images from the missing hand images similarly. However, a one-way ANOVA of c criterion values, a measure of bias, revealed significant differences between groups (F(2,54)=3.31, *p* = 0.04; see Figure S4B). While controls showed no bias (c was close to 0), congenital one handers’ criterion was significantly greater than controls (t(36)=-2.59, *p* = 0.01) suggesting that congenitals have a bias to assume images are of their missing hand side, while amputees were not significantly different from controls in their criterion (t)35)=-1.41, p = 0.17).

### 3.2 Reaction times

The rmANCOVA of reaction times confirmed previous observations (Parsons, 1987, 1994), with a main effect of difficulty with faster RTs for easy vs hard postures across hands and groups (F(1,50) = 18.30, *p* < 0.001). Second, supporting our hypothesis that limited visual and motor experience should result in a difference in RTs across the three patient groups, our analysis showed a significant interaction between difficulty and group (F(2,50)=4.70, p=0.014) and a significant group effect (F(2,50)=3.23, p=0.048). The three-way interaction of group, difficulty and hand was not significant (F(2,50)=1.63, p=0.21; see Supplementary Table S2 for a full description of all other nonsignificant results), indicating that changed performance did not depend on the laterality of the hand, with respect to the amputation/missing hand side.

To further explore the group by difficulty interaction, we ran a post-hoc analysis using a one-way ANCOVA (with group as a fixed effect and age as a covariate). This analysis showed that group differences were significant for hard postures (F(2,50) = 3.77, p = 0.03), but only borderline significant in easy postures (F(2,50) = 2.79, p = 0.07). These results indicate that hand-loss impacts performance on the task, particularly for difficult postures, but independent of the laterality of the presented hand (Fig. 1B&C, left). Exploring the effect of group in RTs of hard posture even further, we found significant differences between congenital one-handers and the other two groups (F(1,51) = 4.62, *p=*0.036), further reflected in a significant pairwise difference between congenital one-handers and the amputees group alone (F(1,30) = 7.62, *p* = 0.01), but not for congenital one-handers compared to controls (F(1,35)=1.6, *p* = 0.21). Importantly, no RT differences were found when we tested a subset of our participants (43 out of 54) on a control visual category naming task involving the same setup, confirming no general deficits in verbal RT for congenital one-handers (BF10= 0.18; using a Bayesian ANCOVA; see Supplementary Methods and Figure S2).

We also explored the present data to identify consistency with previous reports for RT differences in congenital one-handers for judging laterality for intact versus missing hands (Funk & Brugger, 2008). A one-sample *t*-test confirmed a replication of these previous findings, with congenital one-handers performing significantly more slowly when presented with images of their missing hand, compared to their intact hand (t(16)=-3.028, p = .008).

### 3.3 Correlation with phantom hand motor control and amputation age

**Fig. 2:**
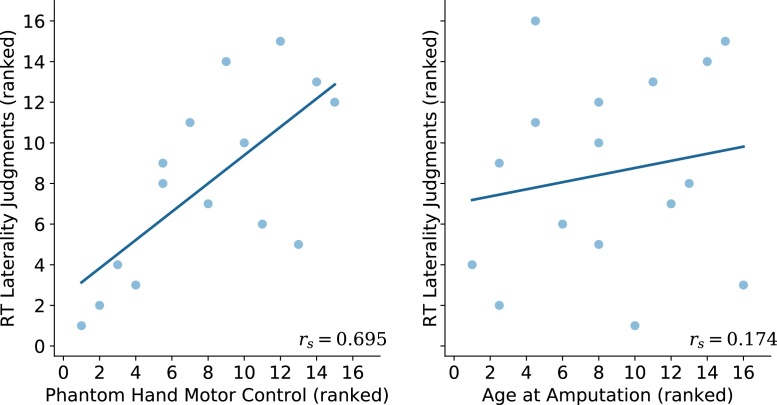
Amputees phantom hand motor control correlated with hand laterality judgement performance. Mean RT (ranked) for laterality judgements (both intact and missing hand images) is significantly correlated to RTs (ranked) in the phantom hand motor control, r_s_(13) = 0.695, p = 0.004. This suggests that existing motor control, rather than visual experience, relates to laterality judgements. Smaller values on the phantom motor task denote faster RTs while executing sequential finger-thumb oppositions with the phantom hand. Age at amputation does not correlate with RT for laterality judgements, r_s_(14) = 0.174, p = 0.52. A direct comparison between the correlation revealed there is a significant difference between the two correlations. Thus, for amputees, motor control of the phantom hand is a better predictor of performance on the laterality task than the lack of visual experience of the missing hand.

To determine the unique role of present non-visual motor control, we correlated amputees’ phantom hand RT during a motor finger-thumb opposition task [as validated in (Kikkert et al., 2017)] with mean RT in the hand laterality judgement task. To explore the role of past active visuomotor experience on hand laterality judgments, we also correlated amputees’ age at amputation (reflecting the amount of time individuals had to accumulate visuomotor hand experience) with hand laterality judgement performance. We found a strong correlation between hand laterality performance and phantom hand motor control (r_s_(13) = 0.695, p = 0.004, Fig. 2), but not with age at amputation (r_s_(14) = 0.174, p = 0.52, Fig. 2; see Supplementary Figure S4 for similar results using RTs for intact and missing hand images separately). This result indicates that better motor control of the phantom hand relates to faster hand laterality judgements. The correlation of phantom motor control with RTs of laterality judgments also remained significant after accounting for participants’ tapping RT with their intact hand (see Supplementary Results). Since amputees’ phantom experience doesn’t have a visual domain, we consider this as strong evidence towards the involvement of motor representation in hand laterality judgments. Furthermore, present motor experience (phantom motor control) was found to be a better predictor for laterality judgement performance than previous active visuomotor experience (age at amputation), using a difference test between two dependent correlations with one variable in common (Z = 1.8, p = 0.036).

Finally, as an exploratory analysis, we also examined the potential link between prosthesis usage (Guo et al., 2017; Nico et al., 2004) and RT performance. We found no correlation between prosthesis usage and mean RT on the laterality task for all study participants with a missing hand (r_s_(31) = .018, p = 0.922), the amputee group alone (r_s_(14) = -.285, p = 0.303) or the congenital group alone (r_s_(15) = -.016, p = 0.953). These findings suggest that prosthesis usage does not strongly inform task performance.

### 3.4 Computational model simulation

To test our interpretation of the potential advantages of parallel bimanual processing during visual laterality judgements (see Discussion), we constructed a post-hoc exploratory simulation, designed to describe a single mechanism that results in the observed error and RT differences between congenital one-handers and controls. We implemented two simultaneous posture simulation processes (one for the left and one for the right hand in controls) to determine whether the seen visual hand posture could be generated using the simulated hand. Conversely, congenital one-handers who only have active visuomotor experience from one hand will deploy a single hand posture simulator. For simplicity, acquired amputees were not included in this simulation, as the model aims to represent a mean observer from each group, and the nature and quality of phantom hand motor control varies across amputees. Moreover, since amputees have residual motor control, they do not provide a compelling test-case for this specific model, beyond the control group.

**Fig. 3:**
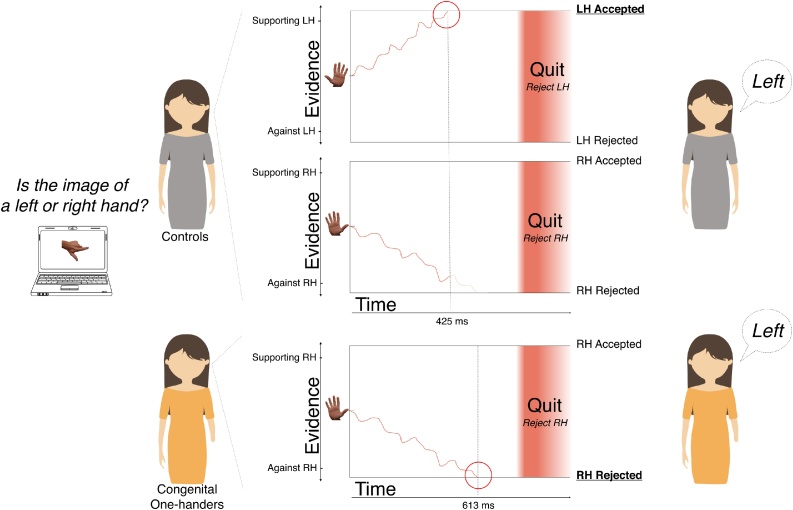
A schematic diagram illustrating the laterality decision making process as simulated by our computational approach. The top panel illustrates a decision process example in a two-handed control (depicted as the woman in the grey dress). In response to the hand image stimulus, left- and right-hand posture models are simultaneously activated, each collecting evidence to either accept or reject whether the hand posture in the stimulus could be generated with that hand. In the example above, once enough evidence is collected by the left-hand posture model to accept that the visual hand posture can be generated by the left hand, the two-handed individual (correctly) judges the hand stimuli as a left hand. Because the actual stimulus is left, the drift rate is positive and negative for the left- and right-hand simulators, respectively. Once the left-hand model collected enough evidence to reach the decision threshold, the right-hand model abandons its process. The bottom panel illustrates the process in response to the same visual stimulus in a right-handed congenital (orange dress). In this example, we illustrate how having a single hand posture model can be less efficient and result in slower reaction times. Since this individual never had visuomotor experience of a left hand, we assume this individual can only utilise a single right-hand posture model to judge hand laterality. In the example above, the right-hand model takes longer to reach the decision threshold, to conclude the presented posture cannot be generated with the right hand. Note that if sufficient evidence had not been collected to reach a decision before the quit timer, then a “reject” decision would be forced.

The model consists of hand posture simulators (based on Drift Diffusion Models) with the addition of a timer component (See Fig. 3). Each hand posture simulator represents a single hand, and accumulates either positive evidence confirming that the posture displayed in the image can be replicated with its hand or negative evidence rejecting that possibility with a drift rate of 0.1. Evidence is accumulated until a threshold is reached, indicating satisfactory information has been gathered to make a decision. Each hand posture model has a starting position between the two thresholds. To generate the slight hand effect [showing faster RT for the intact/dominant hand (Parsons, 1987)], a bias (z = 0.006) was set towards the intact/dominant-hand in both hand posture simulator (i.e. an equal negative bias was set in the non-dominant hand posture simulator). Since some postures might be particularly awkward or difficult to replicate, a timer was added to avoid conceptually long exhaustive evidence accumulations. If the quit-time elapses before enough evidence was accumulated to reach a decision, the hand posture model returns a ‘reject’ decision, meaning it has concluded that the posture cannot be replicated with its hand. In other words, we allow a hand posture model to say: ‘I give up, it’s not this hand’. For each trial a quit-time is drawn from a logarithmic distribution with a mean of 1.6 s and a sigma of 1 s. RT is determined by the time it took the hand posture simulator to reach a decision with the addition of a non-decision time ( = 0.3 s) to account for early-processing and response-generation processes. Finally, since both groups are working under the same time pressure (dictated by the quit timer), and since congenital one-handers are slower in their RT, to maintain the same level of accuracy they would need to lower their evidence threshold (controls-threshold = .17 and congenitals-threshold = .138). This strategy adjustment was made to allow the congenital one-handers to cope with the single simulator situation.

In controls, the model consists of two hand posture simulators, one for each hand, gathering evidence in parallel on whether the viewed hand posture in each trial is of the hand they represent or not. The model that reaches a decision first will determine the trial’s response (the identity of the displayed hand) and RT. In congenital one-handers, only a single hand posture simulator for their intact hand is present. Here, a “dominant-hand” response (e.g. right hand in the example in Fig. 3 in main text) will be the result of the right-hand posture model accumulating evidence in support of the stimulus being of a right hand. While a “nondominant-hand” response will either be: (1) a result of evidence accumulated against the right-hand until rejecting the possibility the viewed posture can be replicated with the right-hand, thus responding “left-hand”. Or (2) accumulating evidence without crossing the evidence threshold, reaching the quit-time, and rejecting the right-hand hypothesis. The simulation has successfully replicated both RT and accuracy group patterns observed in the empirical group results (see pink diamonds in Fig. 1B).

## 4 Discussion

It has been suggested by philosophers that our interactions with the environment may play a fundamental role in the development of our perceptual and cognitive abilities. According to this premise, termed ‘embodied cognition’, body representation may play an important role in shaping cognition. While this view is gaining increasing popularity both in philosophy (Shapiro, 2019), psychology (Fossataro et al., 2018; Garbarini & Adenzato, 2004; Martinaud, Besharati, Jenkinson, & Fotopoulou, 2017; Wilson, 2002) and even in engineering (Metta, Sandini, Vernon, Natale, & Nori, 2008), it still awaits further empirical evidence. In particular, previous studies highlighted the influence of motor disorders on performance on the hand laterality judgement task (2010, Conson et al., 2013; Fiorio et al., 2006; Helmich et al., 2007; Nico et al., 2004). However, this interpretation was confounded by the ecological pairing of visual and motor experience. As such, an alternative interpretation highlighting the reliance on visual experience (Vannuscorps & Caramazza, 2015, 2016; Vannuscorps et al., 2012), and in particular first-person (active) visual experience, cannot be ruled out based on existing evidence. In the present study, we aimed to disentangle the potential contributions of active (self) motor experience from passive visual experience (of others), by studying three groups of individuals with inherently different hand experiences (see Table 1). Our experimental design, involving one-handers with congenital and acquired hand-loss, combined with an evaluation of phantom hand motor control, allowed us to demonstrate that current motor control is a driver in making bodily judgments.

First, we show that congenital one-handers make increased judgment errors for images of their intact hand compared to their missing hand. This counterintuitive finding was previously observed in three congenital one-handers tested in Nico et al. (2004). However, due to the limited sample size, the authors were unable to interpret this finding. Additionally, we show that individuals born with one hand are less effective at judging difficult hand postures’ laterality, in terms of RT profile, than individuals who lost a hand in adult life. The significant difference from amputees rules out the contribution of current visual information on hand laterality judgements and supports a role of either previous visuomotor experience or current motor experience on task performance.

Our reported correlation of amputees’ phantom hand motor control, and not duration of experience with a limb (age at amputation), with visual laterality task performance, provides strong evidence for the role of currently available motor resources when making bodily judgments, supporting the notion of ‘embodied cognition’. Phantom hand motor control has been shown to rely on preserved neural resources across the sensorimotor system (Bruno, Ronga, Fossataro, Capozzi, & Garbarini, 2019; Bruurmijn, Pereboom, Vansteensel, Raemaekers, & Ramsey, 2017; Garbarini, Bisio, Biggio, Pia, & Bove, 2018; Kikkert et al., 2016; Raffin, Richard, Giraux, & Reilly, 2016; Raffin, Mattout et al., 2012; Reilly et al., 2006), and the task used in the current study has been previously related to persistent sensorimotor activity for the missing hand at the peripheral (Raffin, Giraux et al., 2012) and cortical levels (Kikkert et al., 2017). As phantom movements have no visual attributes, studying motor control of the phantom hand provides a unique opportunity to examine the specific contributions of motor processing. Furthermore, our findings also demonstrate that phantom hand motor representation, previously considered as a remnant of the sensorimotor system (Kikkert et al., 2016), potentially related to phantom limb pain (Kikkert et al., 2018, 2017) can be actively utilised to inform body-representation judgements.

The present results showing divergence in hand representation between congenital one-handers and amputees, which showed similar performance to controls, and the suggested role for motor representation in accounting for these differences, is consistent with recent research into brain plasticity subsequent to hand loss. The few functional MRI studies considering plasticity in visual hand representation so far reported no differences between congenital one-handers and acquired amputees (van den Heiligenberg et al., 2018), and between individuals with congenital hand loss and controls (Maimon-Mor et al., 2017; Striem-Amit et al., 2017). This is likely related to the fact that even when considering hand-selective areas, the visual cortex in individuals with a missing hand is not deprived in a strong sense. Conversely, when studying hand representation in primary somatosensory and motor cortex, multiple recent evidence demonstrates that amputees (for review see Makin & Bensmaia, 2017), but not congenital one-handers (Wesselink et al., 2019), show typical sensorimotor representation of their missing hand despite amputation. Instead, congenital one-handers show robust remapping of multiple body-part representations into their missing hand sensorimotor area (Hahamy & Makin, 2018; Hahamy et al., 2017, 2015; also see Striem-Amit, Vannuscorps, & Caramazza, 2018 for evidence of cortical remapping in individuals born without both hands). The extent of this remapping spans well beyond other recent reports for remapping in acquired amputees, which has been shown to be mostly restricted to the intact hand (Makin et al., 2013; Philip & Frey, 2011; Raffin et al., 2016). Collectively, this evidence indicates that amputees have more neural resources than congenital one-handers for updating sensorimotor, but not visual, hand representation, which could contribute to the group similarities and differences in visual laterality judgement revealed in the current study.

As mentioned above, congenitals diverged from the other two groups in terms of error rates, and under some circumstances (particularly when judging challenging hand postures) also showed increased reaction times (e.g. compared with controls and acquired amputees put together). Previous studies demonstrated that congenital one-handers were slower and less accurate bilaterally in a motor-planning task, compared with two-handed controls (Philip et al., 2015), while amputees showed no significant differences from controls (Philip & Frey, 2011). An interesting possible interpretation for the diverging response patterns in congenital one-handers compared to controls and acquired amputees is that they only have a single intact-hand motor representation compared to two hand representations in controls. This account stems from contemporary views of motor planning and action selection (Cisek, 2007; Cisek & Kalaska, 2010; Klein-Flügge & Bestmann, 2012; Oliveira, Diedrichsen, Verstynen, Duque, & Ivry, 2010), where each potential action is refined simultaneously until enough evidence is collected in favour of one action over the others (Cisek & Pastor-Bernier, 2014). To explore this interpretation, we constructed a computational simulation for the congenital one-handers group and control group (see Fig. 3). The simulation has successfully replicated both accuracy and RT group patterns observed in the empirical group results (see pink diamonds in Fig. 1B). The surprising effect of lower accuracy for congenital one-handers intact hand is a result of a single simulator with a single quit timer, generating overall more “missing hand” than “intact hand” responses. In controls, having two simulators means similar likelihood for each to reach the quit time, resulting in a more balanced laterality accuracy pattern. The slower (albite non-significantly so) RT effect with congenital one-handers was induced due to the existence of two parallel simulators in controls vs one in congenital one-handers: since the decision is made by the simulator that reaches the decision threshold first, having twice as much evidence will speed up the convergence on a decision in controls. As such, the number of hand posture models can potentially account for the observed differences between controls and congenital one-handers in the laterality judgement task. We therefore suggest that a bimanual sensorimotor system is more effective than a unimanual system in visual bodily judgements.

In our computational simulation, we equipped the two accumulators for the control group with identical parameters, discounting the potential role of handedness in informing motor decisions (de Lange, Helmich, & Toni, 2006; Gentilucci, Daprati, & Gangitano, 1998; Sekiyama, 1982; Takeda, Shimoda, Sato, Ogano, & Kato, 2010). However, previous studies suggesting that amputation impairs performance on visual laterality tasks were carried in amputees missing their (formerly dominant) right hand (Guo et al., 2017; Guo, Lyu, Bekrater-Bodmann, Flor, & Tong, 2015; Lyu, Guo, Bekrater-Bodmann, Flor, & Tong, 2017; Lyu, Guo, Bekrater-Bodmann, Flor, & Tong, 2016), with amputees with non-dominant hand amputation showing no differences in performance compared to able-bodied controls (Nico et al., 2004). In the present study, we prioritised recruiting amputees missing their left hand (to control for missing hand side in congenital one-handers, which is predominantly left), resulting in a majority of amputees missing their non-dominant hand (n = 12). As such we are unable to comment on the role of motor laterality (as induced by either hand dominance or physical side) on the process of visual laterality judgements. Therefore, our preliminary simulation is exploratory, and awaits further confirmatory evidence and refinement.

In the present study, we focused our investigation on the relationship between visual laterality judgement and preserved motor control, though other factors might also be important contributors to laterality performance. For example, previous evidence is conflicting as to the impact of prosthesis usage on visual laterality judgements of simple postures. Nico et al. (2004) first showed that amputees that regularly wear a prosthesis had decreased performance (both in terms of RT and accuracy) compared to amputees that do not wear a prosthesis and able-bodied controls. Alternatively, Guo et al., 2017 reports an opposite result suggesting that prosthesis usage actually ‘normalises’ the motor body representation (body schema) and therefore facilitates RT in the laterality task (see Schwoebel & Coslett, 2005 for evidence in stroke patients that characterises multiple, distinct body representations, see Heed and Röder (2012) for a discussion of the role of the body schema in multisensory processing, and de Vignemont (2010) for a conceptual analysis of the body schema and the body image). Unlike the simplistic postures of the hand image stimuli used by Nico, Guo et al., prostheses do not afford the complex hand configurations we explore in the present study’s stimuli set. Therefore, prosthesis usage does not necessarily provide useful information for the present task, potentially explaining the lack of correlation between prosthesis usage scores and mean RT on the laterality task for all our study’s participants with a missing hand or the congenital and amputee groups alone.

Put together, we offer several evidences for impaired visual laterality judgement in individuals with congenital hand loss, as summarised in our signal detection analysis and computational simulation. This converging evidence is at odds with recent results of ‘typical’ bodily judgment performance in individuals born without both hands (Vannuscorps & Caramazza, 2015, 2016; Vannuscorps et al., 2012). As mentioned in the introduction, these previous studies provided evidence for convergent processing of visual hand information independent of congenital hand loss, paving the way to the view that visual body representation can be entirely divorced from motor experience. One potential explanation of this conflict is that passive observation alone might be enough to develop a limited model of the biomechanical properties of hands, but this model is insufficient to support an extended repertoire of motor control. For this reason, the stimuli used in our study portrayed high biomechanical complexity, also involving postures and angles that are not typically available through passive visual experience. Another possible reason for the discrepancies between the results in congenital one-handers compared to the bilateral dysmelic individuals is the limited sample sizes used in the latter studies (n = 1–5), resulting in low statistical power, used sometimes in support of the null hypothesis.

In summary, visual information is known to be an influential driver for informing and guiding motor control, as shown in behavioural studies (Honda, Hirashima, & Nozaki, 2012; Saunders, 2004), computational models (Körding & Wolpert, 2004) and in clinical populations (Archer et al., 2018). In the present study, we explored the driving role of motor experience in shaping visual perception and cognition. Specifically, we aimed to gain a better understanding of the role of motor representation in influencing visual bodily judgments. We propose that, when available, motor hand representation is a resource used together with the visual system to optimise hand laterality judgments. Our results provide a novel framework for the process of ‘embodied cognition’, which should be considered in future endeavours for creating neurocognitive-inspired artificial motor or visual systems.

## Declaration of Competing Interest

The authors declare no conflict of interest.

## References

[bib0005] Aglioti S.M., Cesari P., Romani M., Urgesi C. (2008). Action anticipation and motor resonance in elite basketball players. Nature Neuroscience.

[bib0010] Archer D.B., Kang N., Misra G., Marble S., Patten C., Coombes S.A. (2018). Visual feedback alters force control and functional activity in the visuomotor network after stroke. NeuroImage: Clinical.

[bib0015] Bruno V., Ronga I., Fossataro C., Capozzi F., Garbarini F. (2019). Suppressing movements with phantom limbs and existing limbs evokes comparable electrophysiological inhibitory responses. Cortex.

[bib0020] Bruurmijn M.L.C.M., Pereboom I.P.L., Vansteensel M.J., Raemaekers M.A.H., Ramsey N.F. (2017). Preservation of hand movement representation in the sensorimotor areas of amputees. Brain.

[bib0025] Cisek P. (2007). Cortical mechanisms of action selection: The affordance competition hypothesis. Philosophical Transactions of the Royal Society B: Biological Sciences.

[bib0030] Cisek P., Kalaska J.F. (2010). Neural mechanisms for interacting with a world full of action choices. Annual Review of Neuroscience.

[bib0035] Cisek P., Pastor-Bernier A. (2014). On the challenges and mechanisms of embodied decisions. Philosophical Transactions of the Royal Society B: Biological Sciences.

[bib0040] Conson M., Mazzarella E., Frolli A., Esposito D., Marino N., Trojano L., Grossi D. (2013). Motor imagery in asperger syndrome: Testing action simulation by the hand laterality task. PloS One.

[bib0045] Conson M., Pistoia F., Sarà M., Grossi D., Trojano L. (2010). Recognition and mental manipulation of body parts dissociate in locked-in syndrome. Brain and Cognition.

[bib0050] Cooper L.A., Shepard R.N. (1975). Mental transformation in the identification of left and right hands. Journal of Experimental Psychology: Human Perception and Performance.

[bib0055] de Lange F.P., Helmich R.C., Toni I. (2006). Posture influences motor imagery: An fMRI study. NeuroImage.

[bib0060] de Vignemont F. (2010). Body schema and body image-Pros and cons. Neuropsychologia.

[bib0065] Fiorio M., Tinazzi M., Aglioti S.M. (2006). Selective impairment of hand mental rotation in patients with focal hand dystonia. Brain.

[bib0070] Fossataro C., Bruno V., Gindri P., Pia L., Berti A., Garbarini F. (2018). Feeling touch on the own hand restores the capacity to visually discriminate it from someone else’ hand: Pathological embodiment receding in brain-damaged patients. Cortex.

[bib0075] Fozard J.L., Vercruyssen M., Reynolds S.L., Hancock P.A., Quilter R.E. (1994). Age differences and changes in reaction time: The baltimore longitudinal study of aging. Journal of Gerontology.

[bib0080] Funk M., Brugger P. (2008). Mental rotation of congenitally absent hands. Journal of the International Neuropsychological Society.

[bib0085] Garbarini F., Adenzato M. (2004). At the root of embodied cognition: Cognitive science meets neurophysiology. Brain and Cognition.

[bib0090] Garbarini F., Bisio A., Biggio M., Pia L., Bove M. (2018). Motor sequence learning and intermanual transfer with a phantom limb. Cortex.

[bib0095] Gentilucci M., Daprati E., Gangitano M. (1998). Implicit visual analysis in handedness recognition. Consciousness and Cognition.

[bib0100] Gentilucci M., Daprati E., Gangitano M. (1998). Right-handers and left-handers have different representations of their own hand. Cognitive Brain Research.

[bib0105] Green D.M., Swets J.A. (1966). Signal detection theory and psychophysics.

[bib0110] Guo X., Lin Z., Lyu Y., Bekrater-Bodmann R., Flor H., Tong S. (2017). The effect of prosthesis use on hand mental rotation after unilateral upper-limb amputation. IEEE Transactions on Neural Systems and Rehabilitation Engineering.

[bib0115] Guo X., Lyu Y., Bekrater-Bodmann R., Flor H., Tong S. (2015). Handedness change after dominant side amputation: Evaluation from a hand laterality judgment task. Proceedings of the Annual International Conference of the IEEE Engineering in Medicine and Biology Society, EMBS, 2015-Novem.

[bib0120] Hagura N., Haggard P., Diedrichsen J. (2017). Perceptual decisions are biased by the cost to act. ELife.

[bib0125] Hahamy A., Macdonald S.N., van den Heiligenberg F., Kieliba P., Emir U., Malach R., Makin T.R. (2017). Representation of multiple body parts in the missing-hand territory of congenital one-handers. Current Biology.

[bib0130] Hahamy A., Makin T.R. (2018). Reorganization in cerebral and cerebellar cortices is not restricted by proximity between body-part representations. BioRxiv.

[bib0135] Hahamy A., Sotiropoulos S.N., Slater D.H., Malach R., Johansen-Berg H., Makin T.R. (2015). Normalisation of brain connectivity through compensatory behaviour, despite congenital hand absence. ELife.

[bib0140] Heed T., Röder B. (2012). The neural bases of multisensory processes. https://www.ncbi.nlm.nih.gov/books/NBK92834/.

[bib0145] Helmich R.C., de Lange F.P., Bloem B.R., Toni I. (2007). Cerebral compensation during motor imagery in Parkinson’s disease. Neuropsychologia.

[bib0150] Honda T., Hirashima M., Nozaki D. (2012). Adaptation to visual feedback delay influences visuomotor learning. PloS One.

[bib0155] Ionta S., Fourkas A.D., Fiorio M., Aglioti S.M. (2007). The influence of hands posture on mental rotation of hands and feet. Experimental Brain Research.

[bib0160] Kikkert S., Johansen-Berg H., Tracey I., Makin T.R. (2018). Reaffirming the link between chronic phantom limb pain and maintained missing hand representation. Cortex.

[bib0165] Kikkert S., Kolasinski J., Jbabdi S., Tracey I., Beckmann C.F., Johansen-Berg H., Makin T.R. (2016). Revealing the neural fingerprints of a missing hand. ELife.

[bib0170] Kikkert S., Mezue M., Henderson Slater D., Johansen-Berg H., Tracey I., Makin T.R. (2017). Motor correlates of phantom limb pain. Cortex.

[bib0175] Klein-Flügge M.C., Bestmann S. (2012). Time-dependent changes in human corticospinal excitability reveal value-based competition for action during decision processing. The Journal of Neuroscience.

[bib0180] Körding K.P., Wolpert D.M. (2004). Bayesian integration in sensorimotor learning. Nature.

[bib0185] Lee I.A., Preacher K.J. (2013). Calculation for the test of the difference between two dependent correlations with one variable in common.

[bib0190] Lindeløv J.K. (2011). Calculating d’, beta, c and Ad’ in Python and PHP – Neuroscience, stats, and coding.

[bib0195] Lyu Y., Guo X., Bekrater-Bodmann R., Flor H., Tong S. (2016). Phantom limb perception interferes with motor imagery after unilateral upper-limb amputation. Scientific Reports.

[bib0200] Lyu Y., Guo X., Bekrater-Bodmann R., Flor H., Tong S. (2017). An event-related potential study on the time course of mental rotation in upper-limb amputees. Clinical Neurophysiology.

[bib0205] Maimon-Mor R.O., Johansen-Berg H., Makin T.R. (2017). Peri-hand space representation in the absence of a hand – Evidence from congenital one-handers. Cortex.

[bib0210] Makin T.R., Bensmaia S.J. (2017). Stability of sensory topographies in adult cortex. Trends in Cognitive Sciences.

[bib0215] Makin T.R., Cramer A.O., Scholz J., Hahamy A., Slater D.H., Tracey I., Johansen-Berg H. (2013). Deprivation-related and use-dependent plasticity go hand in hand. ELife.

[bib0220] Makin T.R., Wilf M., Schwartz I., Zohary E. (2010). Amputees “neglect” the space near their missing hand. Psychological Science.

[bib0225] Martinaud O., Besharati S., Jenkinson P.M., Fotopoulou A. (2017). Ownership illusions in patients with body delusions: Different neural profiles of visual capture and disownership. Cortex.

[bib0230] Metta G., Sandini G., Vernon D., Natale L., Nori F. (2008). The iCub humanoid robot: An open platform for research in embodied cognition. Proceedings of the 8th Workshop on Performance Metrics for Intelligent Systems - PerMIS’ 08.

[bib0235] Moran R., Zehetleitner M., Liesefeld H.R., Müller H.J., Usher M. (2016). Serial vs. Parallel models of attention in visual search: Accounting for benchmark RT-distributions. Psychonomic Bulletin & Review.

[bib0240] Moseley G.L. (2004). Why do people with complex regional pain syndrome take longer to recognize their affected hand?. Neurology.

[bib0245] Ní Choisdealbha Á., Brady N., Maguinness C. (2011). Differing roles for the dominant and non-dominant hands in the hand laterality task. Experimental Brain Research.

[bib0250] Nico D., Daprati E., Rigal F., Parsons L., Sirigu A. (2004). Left and right hand recognition in upper limb amputees. Brain.

[bib0255] Oliveira F.T.P., Diedrichsen J., Verstynen T., Duque J., Ivry R.B. (2010). Transcranial magnetic stimulation of posterior parietal cortex affects decisions of hand choice. Proceedings of the National Academy of Sciences of the United States of America.

[bib0260] Parsons L.M. (1987). Imagined spatial transformations of one’s hands and feet. Cognitive Psychology.

[bib0265] Parsons L.M. (1994). Temporal and kinematic properties of motor behavior reflected in mentally simulated action. Journal of Experimental Psychology.

[bib0270] Philip B.A., Buckon C., Sienko S., Aiona M., Ross S., Frey S.H. (2015). Maturation and experience in action representation: Bilateral deficits in unilateral congenital amelia. Neuropsychologia.

[bib0275] Philip B.A., Frey S.H. (2011). Preserved grip selection planning in chronic unilateral upper extremity amputees. Experimental Brain Research.

[bib0280] Raffin E., Giraux P., Reilly K.T. (2012). The moving phantom: Motor execution or motor imagery?. Cortex.

[bib0285] Raffin E., Mattout J., Reilly K.T., Giraux P. (2012). Disentangling motor execution from motor imagery with the phantom limb. Brain.

[bib0290] Raffin E., Richard N., Giraux P., Reilly K.T. (2016). Primary motor cortex changes after amputation correlate with phantom limb pain and the ability to move the phantom limb. NeuroImage.

[bib0295] Ratcliff R., Mckoon G. (2008). The diffusion decision model: Theory and data for two-choice decision tasks. Neural Computaion.

[bib0300] Reilly K.T., Mercier C., Schieber M.H., Sirigu A. (2006). Persistent hand motor commands in the amputees’ brain. Brain.

[bib0305] Saunders J.A. (2004). Visual feedback control of hand movements. Journal of Neuroscience.

[bib0310] Schwoebel J., Coslett H.B. (2005). Evidence for multiple, distinct representations of the human body. Journal of Cognitive Neuroscience.

[bib0315] Sekiyama K. (1982). Kinesthetic aspects of mental representations in the identification of left and right hands. Perception & Psychophysics.

[bib0320] Shapiro L. (2019). Embodied cognition.

[bib0325] Shenton J.T., Schwoebel J., Coslett H.B. (2004). Mental motor imagery and the body schema: Evidence for proprioceptive dominance. Neuroscience Letters.

[bib0330] Stanislaw H., Todorov N. (1999). Calculation of signal detection theory measures. Behavior Research Methods, Instruments, & Computers.

[bib0335] Striem-Amit E., Vannuscorps G., Caramazza A. (2017). Sensorimotor-independent development of hands and tools selectivity in the visual cortex. Proceedings of the National Academy of Sciences.

[bib0340] Striem-Amit E., Vannuscorps G., Caramazza A. (2018). Plasticity based on compensatory effector use in the association but not primary sensorimotor cortex of people born without hands. Proceedings of the National Academy of Sciences.

[bib0345] Takeda K., Shimoda N., Sato Y., Ogano M., Kato H. (2010). Reaction time differences between left- and right-handers during mental rotation of hand pictures. Laterality.

[bib0350] van den Heiligenberg F.M.Z., Orlov T., MacDonald S.N., Duff E.P., Henderson Slater D., Beckmann C.F., Makin T.R. (2018). Artificial limb representation in amputees. Brain.

[bib0355] van den Heiligenberg F.M.Z., Yeung N., Brugger P., Culham J.C., Makin T.R. (2017). Adaptable categorization of hands and tools in prosthesis users. Psychological Science.

[bib0360] Vannuscorps G., Caramazza A. (2015). Typical biomechanical bias in the perception of congenitally absent hands. Cortex.

[bib0365] Vannuscorps G., Caramazza A. (2016). Typical action perception and interpretation without motor simulation. Proceedings of the National Academy of Sciences.

[bib0370] Vannuscorps G., Pillon A., Andres M. (2012). Effect of biomechanical constraints in the hand laterality judgment task: Where does it come from?. Frontiers in Human Neuroscience.

[bib0375] Wesselink D.B., Heiligenberg F.M., Van Den Ejaz N., Dempsey-Jones H., Cardinali L., Tarall-Jozwiak A., Makin T.R. (2019). Obtaining and maintaining cortical hand representation as evidenced from acquired and congenital handlessness. ELife.

[bib0380] Wilson M. (2002). Six views of embodied cognition. Cognition.

