# Supplementary material for: Motor control drives visual bodily judgements
Source: Cognition. 2020 Mar;196:104120. doi: 10.1016/j.cognition.2019.104120 (PMC7033558; doi:10.1016/j.cognition.2019.104120)
Supplement: Supplementary file 1 [file mmc1.docx]

# Supplementary Material

## Supplementary Methods and Results

### Hand Laterality Judgement: Stimuli Difficulty Split

To asses hand posture image difficulty, a separate group of 13 able-bodied two-handed controls (mean age ± s.e.m. = 42.9 ± 3, 4 left-hand dominant; 3 females) performed the hand laterality judgment task described in the main text with the only exception of completing a single experimental block instead of two. In other words, each hand posture image was presented only once. RTs where averaged across hands (left- and right-hand image of the same posture) and participants. Difficulty was categorised using a median split, with images below the overall median (1.46 sec) labelled as ‘easy’, and above as ‘hard’ (see Figure S1 for all stimuli divided to easy and hard postures).

### Control Task: Category Naming

Data used here was taken from a previously published study with the same participants (van den Heiligenberg, Yeung, Brugger, Culham, & Makin, 2017). In brief, ﻿participants performed a visual priming task in which they verbally categorized target images of hands and tools. The experimental set up was identical to that used in the present study, and data was collected during the same session. Trials included in our analysis are the 40 baseline trials in which a scrambled image (neutral prime) was followed by a target stimulus of a hand or a tool, presented for 32 ms (stimulus onset asynchrony = 600 ms). This task was included to test whether congenitals are generally slower then controls in verbal responses to related visual stimuli regardless of the task, or whether the slowing is specific to the hand laterality judgment task. A subset of 19 two-handed controls, 12 amputees and 12 congenitals participated took part in the category naming task. In an ANCOVA with Age as a covariate no group differences were found (F(2,40)=0.06 p=0.94). A Bayesian ANCOVA performed using JASP v0.9.0.1 (Jasp Team, 2018) revealed a BF < 0.33, supporting the null hypothesis of no performance differences between groups in the control task (BF10= 0.18, BF01=5.43; See Figure S2).

### Correlation with phantom hand motor control in amputees

In the main text, we report a significant correlation between phantom hand motor control (phantom finger tapping response time) and overall mean RT in the hand laterality judgement task. To rule out the possibility that this correlation is driven by general inter-individual differences in speed, we also measured participants’ intact hand motor control (intact hand finger tapping response time) and created a measure of phantom motor control that accounts for intact motor control by using the phantom motor control residuals from the correlation between the two (intact and phantom hand motor control). The residuals, reflecting the unique contribution of phantom hand response times after accounting for inter-individual differences in speed, were then correlated with overall mean RT in the hand laterality judgement task using a Spearman correlation resulting in a significant relationship between phantom hand motor control and visual hand laterality judgements [*r_s_*(13)=0.525, *p*=0.04]. Furthermore, to show that using the overall mean RT for all hand images in the hand laterality task is not driven by RTs for intact or missing hand posture image we’ve correlated each of them separately with phantom hand motor control, resulting in a correlation of r_s_(13) = .665, p = .007 for intact hand images and r_s_(13) = .652, p = .008 for missing hand images (see Figure S3).

## References

Jasp Team. (2018). JASP.

Moran, R., Zehetleitner, M., Liesefeld, H. R., Müller, H. J., & Usher, M. (2016). Serial vs. parallel models of attention in visual search: accounting for benchmark RT-distributions. *Psychonomic Bulletin & Review*, *23*(5), 1300–1315. https://doi.org/10.3758/s13423-015-0978-1

Parsons, L. M. (1987). Imagined spatial transformations of one’s hands and feet. *Cognitive Psychology*, *19*(2), 178–241. https://doi.org/10.1016/0010-0285(87)90011-9

Ratcliff, R., & Mckoon, G. (2008). The Diffusion Decision Model: Theory and Data for Two-Choice Decision Tasks. *Neural Computation*, *20*, 873–922.

van den Heiligenberg, F. M. Z., Yeung, N., Brugger, P., Culham, J. C., & Makin, T. R. (2017). Adaptable Categorization of Hands and Tools in Prosthesis Users. *Psychological Science*, *28*, 395–398. https://doi.org/10.1177/0956797616685869

‘Easy’ Posture Images

‘Hard’ Posture Images

**Figure S1 – Difficulty classifications for laterality hand posture images**. Hand postures were divided to easy and hard based on hand laterality judgement RTs from a separate control group.

**Figure S2 – Control visual category naming task.** RT group performance in the category naming task. Displayed are means ± standard error. No group differences found, as validated using a Bayesian analysis, pointing towards a lack of general cognitive deficit in congenitals relating to the current task setup.

**Figure S3 - Amputees phantom hand motor control correlated with hand laterality judgement performance for each hand side** - (A) mean RT (ranked) for images of the intact hand side in the hand laterality correlated to phantom finger-tapping RT (ranked), r_s_(13) = .665, p = .007, (B) Missing hand RT laterality judgements (ranked) correlated to phantom finger-tapping RT (ranked), r_s_(13) = .652, p = .008.

**Figure S4 – Signal Detection Theory d-prime and c-criterion.** (A) d’ group results, all groups show similar discriminability between intact and missing hand posture images (F(2,54)=0.04, p=.96). (B) Critetion (c) group results, congenitals show a bias to assume images are of their missing hand side (F(2,54)=3.31, p=0.04).

| Effect | df | F | p |
| --- | --- | --- | --- |
| difficulty | 50,1 | 18.296 | <.001 |
| difficulty * age | 50,1 | .024 | .877 |
| difficulty * group | 50,2 | 4.696 | .014 |
| hand | 50,1 | 1.850 | .180 |
| hand * age | 50,1 | .362 | .550 |
| hand * group | 50,2 | .970 | .386 |
| difficulty * hand | 50,1 | .810 | .373 |
| difficulty * hand * age | 50,1 | .242 | .625 |
| difficulty * hand * group | 50,2 | 1.632 | .206 |
| age | 50,1 | .500 | .483 |
| group | 50,2 | 3.226 | .048 |

**Table S2 – Results of *reaction time (RT)* rmANCOVA**

| Effect | df | F | p |
| --- | --- | --- | --- |
| difficulty | 50,1 | .203 | .654 |
| difficulty * age | 50,1 | 2.416 | .126 |
| difficulty * group | 50,2 | .988 | .380 |
| hand | 50,1 | .703 | .406 |
| hand * age | 50,1 | .045 | .833 |
| hand * group | 50,2 | 3.920 | .026 |
| difficulty * hand | 50,1 | 1.976 | .166 |
| difficulty * hand * age | 50,1 | .376 | .542 |
| difficulty * hand * group | 50,2 | .863 | .428 |
| age | 50,1 | .000 | .988 |
| group | 50,2 | .292 | .748 |

**Table S3 – Results of *accuracy* rmANCOVA**

| Effect | df | F | p |
| --- | --- | --- | --- |
| difficulty | 50,1 | .377 | .542 |
| difficulty * age | 50,1 | 2.786 | .101 |
| difficulty * group | 50,2 | 1.337 | .272 |
| hand | 50,1 | .627 | .432 |
| hand * age | 50,1 | .077 | .782 |
| hand * group | 50,2 | 3.338 | .044 |
| difficulty * hand | 50,1 | 1.573 | .216 |
| difficulty * hand * age | 50,1 | .310 | .580 |
| difficulty * hand * group | 50,2 | 1.770 | .181 |
| age | 50,1 | .249 | .620 |
| group | 50,2 | 1.341 | .271 |

**Table S4 – Results of RT/accuracy rmANCOVA.** Though note that Box's Test of Equality of Covariance Matrices was significant (Box’s M= 45.09, F(20,8386.36)=1.99, p= 0.005).

A


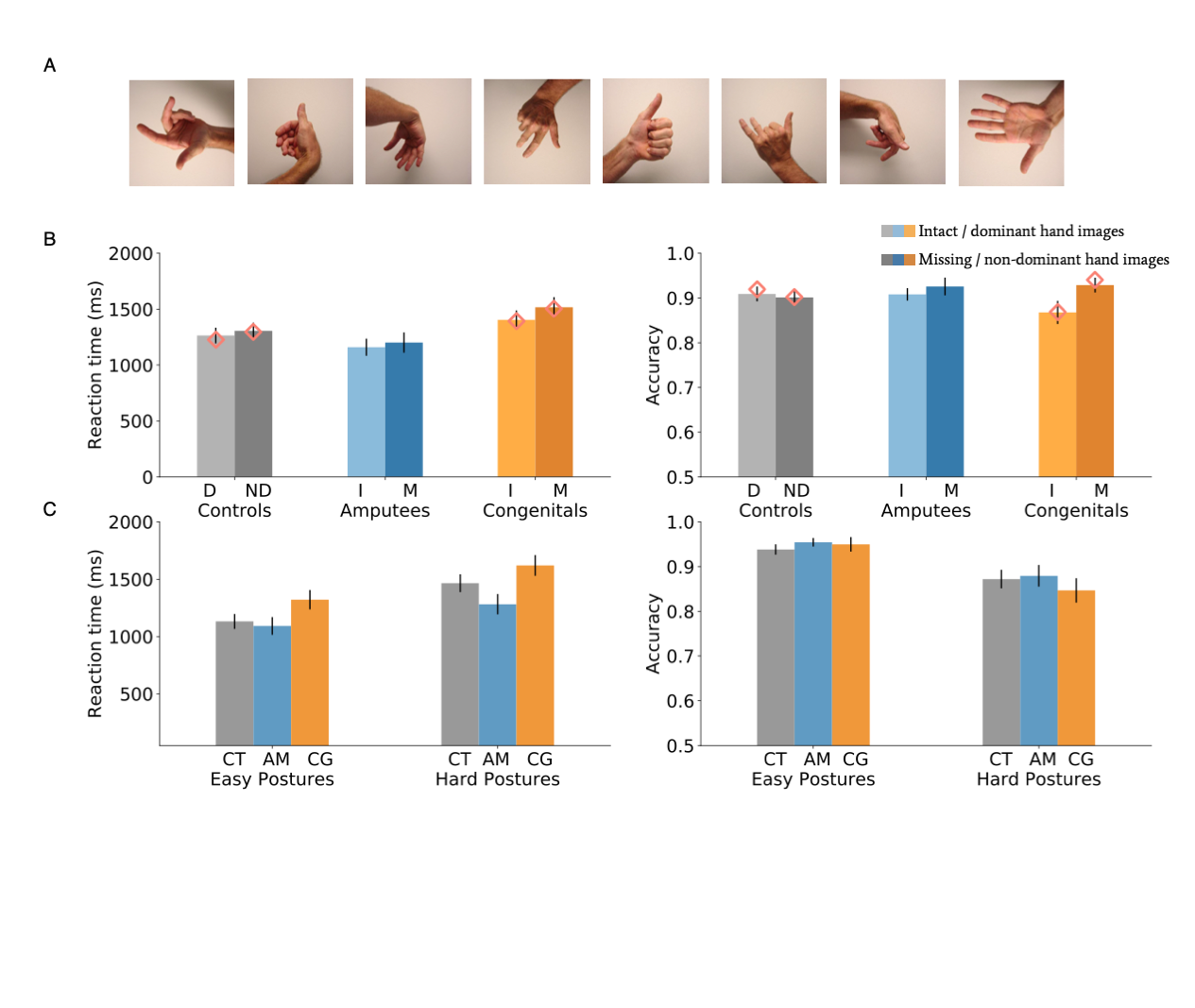


B

**Figure S5 – Log-transformed RT plots.** (A) Group and individual performance (log-tranformed RT values) in the hand laterality judgement task is shown for controls (grey), amputees (blue) and congenital one-handers (orange) for the intact and missing hands (light vs dark shades, respectively). Dots correspond to individual performance. (B) Group performance (log-transformed RT values) in the hand laterality judgement task in the hand laterality judgement task is shown for easy and hard postures in controls (grey), amputees (blue) and congenital one-handers (orange). Displayed are means ± standard error. CT=controls; AM=amputees and CG=congenital one-handers.

|  | Reaction time  (seconds, *mean ± standard error*) | Accuracy  (%, *mean ± standard error)* |
| --- | --- | --- |
| Controls | 1.278 seconds ± .317 | 90.5% ± 6.73 |
| Amputees | 1.176 seconds ± .319 | 91.7% ± 5.69 |
| Congenitals | 1.455 seconds ± .352 | 89.8% ± 8.04 |

**Table S6 – Average group reaction time & accuracy values for all hand images.**
